# Is Your Ethics Committee Efficient? Using *“IRB Metrics”* as a Self-Assessment Tool for Continuous Improvement at the Faculty of Tropical Medicine, Mahidol University, Thailand

**DOI:** 10.1371/journal.pone.0113356

**Published:** 2014-11-18

**Authors:** Pornpimon Adams, Jaranit Kaewkungwal, Chanthima Limphattharacharoen, Sukanya Prakobtham, Krisana Pengsaa, Srisin Khusmith

**Affiliations:** 1 Office of Research Services, Faculty of Tropical Medicine, Mahidol University, Bangkok, Thailand; 2 Department of Tropical Hygiene, Faculty of Tropical Medicine, Mahidol University, Bangkok, Thailand; 3 Department of Tropical Pediatrics, Faculty of Tropical Medicine, Mahidol University, Bangkok, Thailand; 4 Department of Microbiology and Immunology, Faculty of Tropical Medicine, Mahidol University, Bangkok, Thailand; Centre for the AIDS Programme of Research in South Africa (CAPRISA), South Africa

## Abstract

Tensions between researchers and ethics committees have been reported in several institutions. Some reports suggest researchers lack confidence in the quality of institutional review board (IRB) reviews, and that emphasis on strict procedural compliance and ethical issues raised by the IRB might unintentionally lead to delays in correspondence between researchers and ethics committees, and/or even encourage prevarication/equivocation, if researchers perceive committee concerns and criticisms unjust. This study systematically analyzed the efficiency of different IRB functions, and the relationship between efficiency and perceived quality of the decision-making process. The major purposes of this study were thus (1) to use the IRB Metrics developed by the Faculty of Tropical Medicine, Mahidol University, Thailand (FTM-EC) to assess the operational efficiency and perceived effectiveness of its ethics committees, and (2) to determine ethical issues that may cause the duration of approval process to be above the target limit of 60 days. Based on a literature review of definitions and methods used and proposed for use, in assessing aspects of IRB quality, an “IRB Metrics” was developed to assess IRB processes using a structure-process-outcome measurement model. To observe trends in the indicators evaluated, data related to all protocols submitted to the two panels of the FTM-EC (clinical and non-clinical), between January 2010–September 2013, were extracted and analyzed. Quantitative information based on IRB Metrics structure-process-outcome illuminates different areas for internal-process improvement. Ethical issues raised with researchers by the IRB, which were associated with the duration of the approval process in protocol review, could be considered root causes of tensions between the parties. The assessment of IRB structure-process-outcome thus provides a valuable opportunity to strengthen relationships and reduce conflicts between IRBs and researchers, with positive outcomes for all parties involved in the conduct of human-subject research.

## Introduction

The question whether the ethics committee is facilitating or impeding the conduct of research is sometimes hotly debated. Institutional review boards (IRBs) are at times accused of being the “ethics police”, and researchers frequently complain about conflicts and power relationships *vis-a-vis* their IRBs [1]. This poor relationship might potentially result in negative or inadequate outcomes for human-subject protection. Even though both researchers and IRBs agree on the principle of protecting human subjects, some researchers argue that IRB members frequently act beyond the scope of their mandate – the protection of research subjects – and behave paternalistically towards them [2]. Several studies have suggested that researchers lack confidence in the quality of IRB reviews [3], [4], and that the emphasis on strict procedural compliance might encourage prevarication/equivocation on matters where researchers perceive concerns and criticisms as potentially unjust [5], [6]. Some researchers have expressed concern that there is no compilation of rulings and precedents of IRB mandates, so that each newly populated IRB creates many of its own decision-making rules *de novo* [7].

Studies requiring multiple IRB submissions face even more disturbing issues, due to local differences in the implementation of principles, resulting in each research institution creating its own IRB with its own rulings and precedents on protocol review. Indeed, different IRBs within the same institution may reach different rulings on identical protocols. Some researchers even report that IRBs sometimes lack a dualistic perspective and that due to the IRBs’ good intentions towards human subjects, oblige researchers and other IRBs to comply only with their viewpoint. This frustrating experience has been faced by researchers conducting multi-site or multi-country trials [7]–[9]. A survey conducted among 203 researchers in developing countries reported that ethics committees were more concerned with politics than protecting the interests of research subjects [8]. Another study, examining differences in ethical judgments of IRBs across Europe and the United States, reported that only 5 of 26 (19%) reports received unanimous IRB approval. The remaining protocols were rejected by at least one IRB, with different clarifications and revisions being requested [9]. Attempts have been made to use a central IRB to avoid such situations, but there are still several unsettled issues and concerns about the quality and appropriateness of using a central IRB. IRBs and researchers often appear to have divergent thoughts regarding the quality and standards of protocol review. There are unintended consequences, such as discrepancies among IRBs in terms of differences in institutional culture and history, and personalities of chairs and/or more vocal members [10]. A study on barriers to the use of central IRBs for multicenter clinical trials in the USA suggested that the major obstacles were related to conflating responsibilities among institutions with ethical-review responsibilities of their own IRB [11].

There have been different approaches to assessing IRB process quality, and several studies highlight variations among local review processes [12]–[15]. A review of quality assessments of IRB ethical processes suggests that they mostly focus on administrative efficiency and consistency of reviews for multi-site trials. It notes that some studies addressed the ethical component of the IRB review process by looking at the criteria used most often by IRB members to assess the ethical quality of research: scientific merit, balance of risk and benefit, participation information sheet, and informed consent process [16]. Metrics based on the IRB review process have been developed and used as indicators to assess whether its operations have made an impact on human research protection or not. These metrics can be used as baseline information for organizational improvement, and to provide an evidence base for the demonstration of the effectiveness of Ethics Committees related to research subjects, researchers, and research management [17].

Different metrics have been constructed by various institutions to gather quantitative and qualitative information about IRB performance. The Research Compliance Office of Stanford University uses its own metrics and compiles data periodically–analyzing protocol activity reports, and conducting surveys of the research community and IRB members [18]. Other universities have standard operating procedures (SOPs) that govern the use of metrics, or checklists to collect ethics process-quality information: Boston University Medical Campus uses IRB policies and procedures to review, assess, remediate, and improve IRB process quality [19]. The University of Central Florida, USA, uses a checklist on “Minutes Quality Improvement Assessment”, covering items such as number of members attending committee meetings, issues reviewed and discussed, etc [20]. The University of Missouri-Columbia has SOPs on assessments/audits for continued quality improvement by measuring constructive communication with research stakeholders and by identifying barriers to effective compliance [21]. The Mayo Clinic Human Research Protection Program has SOPs on “Roles, Qualifications, and Evaluation of IRB Members” that evaluate convened-IRB members with an “IRB Self-Assessment Form”, to facilitate the annual assessment process [22].

Despite the fact that several IRBs have developed mechanisms or proposed best practices for improving the efficiency of their processes, for assessing their effectiveness and the service levels of their operations, for documenting the rationales for their decisions, and for justifying variations in the review process and outcomes, reports persist of tensions and conflicts between IRBs and researchers [23], [24]. However, few pay systematic attention to whether, how, when, and why IRBs respond to these tensions/conflicts [24]. Lacking a tool to measure the efficiency of IRB reviews, it is difficult to determine whether, and identify which, aspects of IRB functions in relation to the assurance of IRB process efficiency and quality should be of concern [16]. Moreover, in an attempt to reduce potential conflicts between researchers and ethics committees, it would be helpful to have a tool for assessing protocol-review outcomes that might unintentionally lead to delays in correspondence, and resolutions of matters of mutual concern, between researchers and ethics committees. The two major purposes of this study were thus: (1) to use the IRB Metrics developed by FTM-EC as a measurement tool to assess the performance of ethics committees, regarding the efficiency and effectiveness of their operation, and (2) to determine ethical issues related to the quality of research that may cause the duration of the approval process to exceed the target of 60 days.

## Methods

### Ethics Statement

To support, manage, and promote the conduct of research at the Faculty of Tropical Medicine, Mahidol University, the Office of Research Services (ORS) provides administrative services to the faculty’s research community. One of its major functions is to function as the Secretariat to the Faculty’s Ethics Committee, managing the operations of Ethics Committees at the Faculty of Tropical Medicine (FTM), Mahidol University, Thailand. The Faculty of Tropical Medicine Ethics Committee (FTM-EC) has been continuously registered with the Federal-wide Assurance (FWA) of the US-Office for Human Research Protections (OHRP), since 2002. The Ethics Committee is composed of two panels, Panel I (clinical) and Panel II (non-clinical).

### Development of a performance assessment tool

Ethics and quality are intimately related to each other and it would not be an overstatement to argue that quality is an embodiment of ethics [25]. Both concepts can have different meanings for different individuals. An evaluation of the quality of something is unavoidably relative, and it is difficult to reach a consensus on a definition that encapsulates the same meaning for all [25]. One approach divides quality into two categories – one being “objective quality” and the other “subjective quality”, but their definition still varies from person to person [26]. Another approach suggests that quality consists of simultaneously achieving two components, “efficiency” (*doing the right things*) and “effectiveness” (*doing the things correctly*) [27].

It is suggested that without a measure of quality, IRBs, together with other stakeholders (including sponsors, regulators, and the public) would have no evidence-based information from which to draw conclusions about particular human-subject research [16], [28]. However, precisely how quality should best be measured has been hotly debated. Various measures and definitions have been proposed as proxy indicators of IRB quality. Some suggest that an IRB should set up its own measurement parameters to help it determine what is or is not working, and what the current trend is [29]. For this strategy, IRBs should select areas to track and trend, measure error rates, and assess the relationship between staffing levels and workload. Parameters may include time from submission to completion of an IRB review, number of submissions and exemptions, staffing levels, etc. Several institutions and organizations have developed IRB self-assessment tools [30]. A working group of research ethics committees and researchers from the Middle East proposed initial standards that incorporate surrogate metrics considered to be foundational for effective human-subject protection. These include IRB policies, structural elements (e.g., membership composition), and processes and performance standards (e.g., submission of protocols, communicating a decision). The final self-assessment tool of this group is divided into different categories: (a) organizational aspects, (b) membership, (c) submission arrangements, (d) minutes, (e) review procedures, (f) communicating a decision, (g) continuing review, and (h) IRB resources.

The US Food and Drug Administration (US-FDA) has developed a self-evaluation checklist to help IRBs/institutions evaluate procedures for the protection of human research subjects [31]. It suggests that once an IRB/institution has established its structure and procedures, the process/procedural topics in the checklist should be reviewed regularly, and updated as necessary to ensure currency. The checklist consists of several topics, including written policy and procedures, the membership and management of the IRB, the functions and operations of the IRB, communication from the IRB, and record requirements. Similarly, the US Department of Health & Human Services (DHHS), Office for Human Research Protections (OHRP) has “OHRP QA Self-Assessment Tool”, comprising 98 items on general administrative information on the IRB component, workload of the IRB(s) and staffing resources, review and continuing review process, and IRB management and minutes/records [32]. In an attempt to establish mechanisms to regulate and assess the operations and functions of IRBs, IRB registration, coupled with audits and accreditation, was established [33]–[37]. The regulatory authorities have supported the use of accreditation standards to evaluate IRB performance [38].

In the USA, IRBs are usually accredited by the Association for the Accreditation of Human Research Protection Programs (AAHRPP). Evaluation for AAHRPP accreditation focuses on three domains – organization, IRB, and researcher/research staff [39]. The routinely scheduled (usually annual) IRB evaluation is used to validate performance and identify areas needing improvement. The assessments vary from self-assessments to objective and subjective evaluation by peers or supervisors. Measurements include both quantitative and qualitative information, as well as objective and subjective criteria for IRB quality. The objective criteria include number of protocols reviewed by the convened meeting or by expedited procedure, number of exemptions, and number of reviews completed by the primary reviewer. Subjective criteria include leadership of the IRB, preparedness for meetings, relationship between IRB chair and IRB staff, and ability to help investigators. The objective criteria for IRB evaluation include workload, timeliness of processing materials, completion checklists and documentation, and the preparation of convened IRB minutes in a timely manner.

A systematic review of 43 empirical studies was conducted, looking at various aspects of U.S. IRB structure, processes and outcomes, or variations in processes or outcomes among different IRBs using different study methodologies: surveys, analysis of written documents (e.g. IRB minutes), interviews with IRB members, administrators, or investigators, and site visits [23]. The studies in the review were classified according to the main study issues: IRB structure, IRB process, and IRB outcomes. The studies of IRB structure included evaluations of IRB membership characteristics, IRB costs, the volume of studies reviewed, and the experience of nonaffiliated, nonscientific IRB members. The studies of IRB processes included the evaluation of a particular aspect of the review process, reviewing emergency research, and community involvement or consultation. The studies of IRB outcomes included variations in review practices and outcomes, e.g., decisions about compensation, consent/assent process, and risks involved in specimen collection. It was suggested that this systematic review would provide evidence-based information about inconsistencies in the structures, processes, and outcomes of IRB reviews, and would support researcher complaints about IRB inconsistencies, delays, and inefficiencies.

Based on the review above, of methods for assessing IRB quality, the FTM-EC has developed an internal “IRB Metrics” in an attempt to assess the performance of its ethics committee panels. The IRB Metrics adopts three aspects in assessing quality, using structure, process, and outcome measures that have been used in both human-subject and animal ethics reviews [40]–[42]. Like the metrics described by the AAHRPP [43], the purposes of this metric are to provide information to help improve the IRB, to promote the use of quality indicators by the faculty, and for use as benchmarks to compare performance over time, or with other organizations. The indicators of IRB Metrics of FTM-EC, employing the structure-process-outcome model, are summarized in Table 1.

**Table 1:** IRB Metrics structure-process-outcome model used at FTM-EC.

EvaluationApproach	Goals & Performance Measures
Evaluation ofStructure	Evaluation of committee composition, qualification, and workload.
Evaluation ofProcess	Evaluation of review procedures, management convened meetings, decision-making processes (individual and group), variations in time for review, and IRB site-monitoring visits.
Evaluation ofOutcome	Evaluation of review outcomes, approval rates, ethics quality issues raised and communicated to researchers, and researchers’ comments about IRB performance.

### Source of information and statistical analysis

The IRB Metrics has been used as an internal quality-assurance tool for assessing the performance of the FTM-EC. The policies and SOPs are subject to annual review by the Office of Research Services. This study adopts the process of internal audits on quality systems of independent ethics committees in Europe [44], by conducting documentation and process reviews. Documentation review (for structure and outcome) includes a review of documents about IRB members/expert reviewers, minutes of meetings and agendas, files on projects reviewed/approved/declined, the individual IRB member’s review form provided prior to every convened meeting, notifications to researchers, and the annual report of the IRB’s activities. Process review includes a review of meeting times, number of IRB members participating per meeting, timeliness of the approval process, assessment of ethical issues discussed and notified to the researcher and study sites, processes for safety and deviation report reviews, and processes for review of annual continuing studies and amendments.

The information used in the present study was extracted by personnel authorized to access these documents. To avoid bias, three office employees (not voting members of the FTM-EC), were assigned to use the “IRB Metrics” while cross-checking with one another. Almost all indicators in the metrics are quantitative. Issues related to the ethical quality of research that are raised by IRB members (scientific merit, risk and benefit, sample size, vulnerable subject-related issues, or informed-consent process) were collected from the check-list, and open-ended items on each individual IRB member’s review form. Therefore, very little subjective judgment is required by the person extracting the data.

To identify trends in the indicators evaluated, the information was collected over a period of 45 months, from January 2010 to September 2013. The analyses were presented by year, type of FTM-EC ethics committee panel (clinical or non-clinical study), type of review (convened-meeting review or expedited review), and type of study (new non-exempt study or continuing-amended study). To identify issues that could potentially impact approval time and cause processing periods “above the target duration”, the prevalence rate ratio was calculated. The target duration of the approval process, from submission to final approval, was set at ≥60 days.

To examine how well the FTM-EC has been performing, some statistics from 2012 IRB Metrics that are similar to the AAHRPP Metrics have been compared descriptively. For the purpose of benchmarking FTM-EC with other AAHRPP accredited institutes with similar workload, selected comparable indicators in the AAHRPP report that could help identifying the ethics committee’s performing practices were included: types of reviewed research, conformance with regulations and guidance to IRB review times, and IRB review outcomes.

## Results

### IRB Structure

As shown in Table 2, the composition of FTM-EC, under both policy and institutional standard operating procedures (SOPs), has followed international standards. There are 16 members in Panel I, which reviews clinical studies, and 12 in Panel II, which reviews non-clinical (biomedical and observational) studies. The two panels consist of professionals with different areas of expertise, and laypersons (more physicians in Panel I; more scientists in Panel II); the gender distribution is about 60% female: 40% male. All IRB members (average age 54, range 28–69) have many years’ experience working in their respective fields. During a review, all IRB members receive the protocol and an individual review checklist of ethical critical issues (scientific merit, type of study, criteria for human-research-subject study, involvement of vulnerable subjects, sampling techniques, specimen collection, toxicology, qualifications of investigators, budget, facilities, and informed consent process). In addition, each IRB member considers an open-ended assessment form for each part of the protocol. Each protocol is assigned to two or three primary reviewers, who are responsible for reviewing the protocol in detail with a more comprehensive checklist. On average, for 3 clinical and 1 non-clinical studies per month, FTM-EC enlisted external reviewers or consultants with special expertise not available in the EC, to examine the protocols.

**Table 2:** Evaluation of Structure – IRB composition and qualifications.

Metrics	Clinical studies(Panel I)	Non-clinical studies(Panel II)
**IRB Member Characteristics**		
**Numbers of IRB Members by Personal Qualifications**		
**TOTAL**	16	12
**By affiliation:**		
FTM staff	13	8
*-Academic*	10	6
*-Non-academic*	3	2
Non-affiliated to FTM	3	4
**By expertise**		
Physician (MD/MBBS)	9	3
Scientist	3	5
Social scientist	1	0
Statistician	1	1
Lawyer	1	1
Lay person	1	2
**By sex distribution**		
Male	7	5
Female	9	7
**Age**		
Mean (Min–Max)	52 (35–64)	56 (28–69)
**Reviewers of Protocols**(excluding other IRB members who read and commented on protocols)		
**Number of Primary Reviewers per Study**		
Convened review – Average (Min–Max)	2 (2–3)	2 (2–2)
Expedited review – Average (Min–Max)	0	2 (2–2)
**Use of Alternate Reviewers and Consultants (monthly)**		
Number of studies – Average	3	1
Number of reviewers – Average (Min–Max)	2 (1–3)	2 (1–3)
**IRB support staff**		
**Numbers of Staff Working on Pre-review Process**		
Scientific staff	1	1
Administrative Staff	1	1

**Figure 1:**
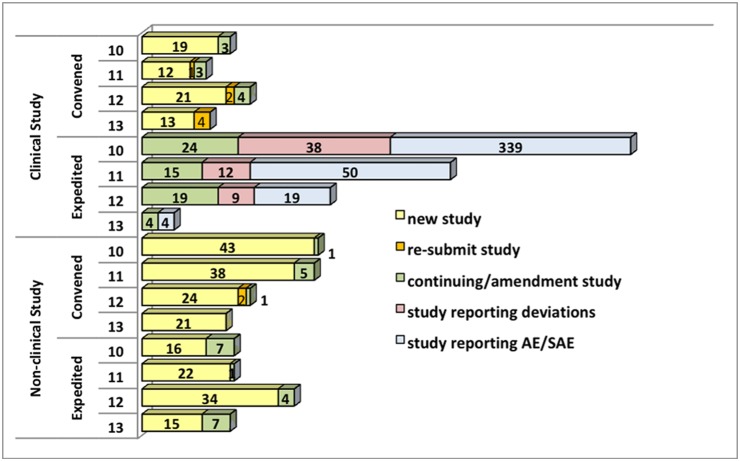
Evaluation of Structure – IRB workload & number of protocols/documents reviewed, 2010–2013.

The workload of the FTM-EC during the study period (45 months, from January 2010 to September 2013) consisted of 278 new non-exempt studies and 98 continuing studies. On average, there were about 37 clinical (47% new and 53% continuing) and 64 non-clinical (89% new and 11% continuing) studies per year. Continuing-study review comprised studies with and without amendments; ones with amendments required a full IRB ethics assessment. Those without amendments require IRB preview staff and designated primary reviewers to read the protocol to cross-check for any changes from the original. Of all studies submitted for IRB review, 14 were subsequently withdrawn – 6 by the investigator due to various internal matters relating to the study team (financial support, change of study site, feasibility of the study), and 8 by the ethics committee due to very long non-response after notification (>6 months). As shown in Figure 1, a few studies were resubmitted (including revised studies terminated or disapproved). At least two IRB members were required to review deviations and adverse events/serious adverse events (SAEs). It should be noted that a decreasing number of studies reported such incidents – particularly studies reporting AE/SAE decreased significantly as a few large-scale clinical trials were completed.

**Figure 2:**
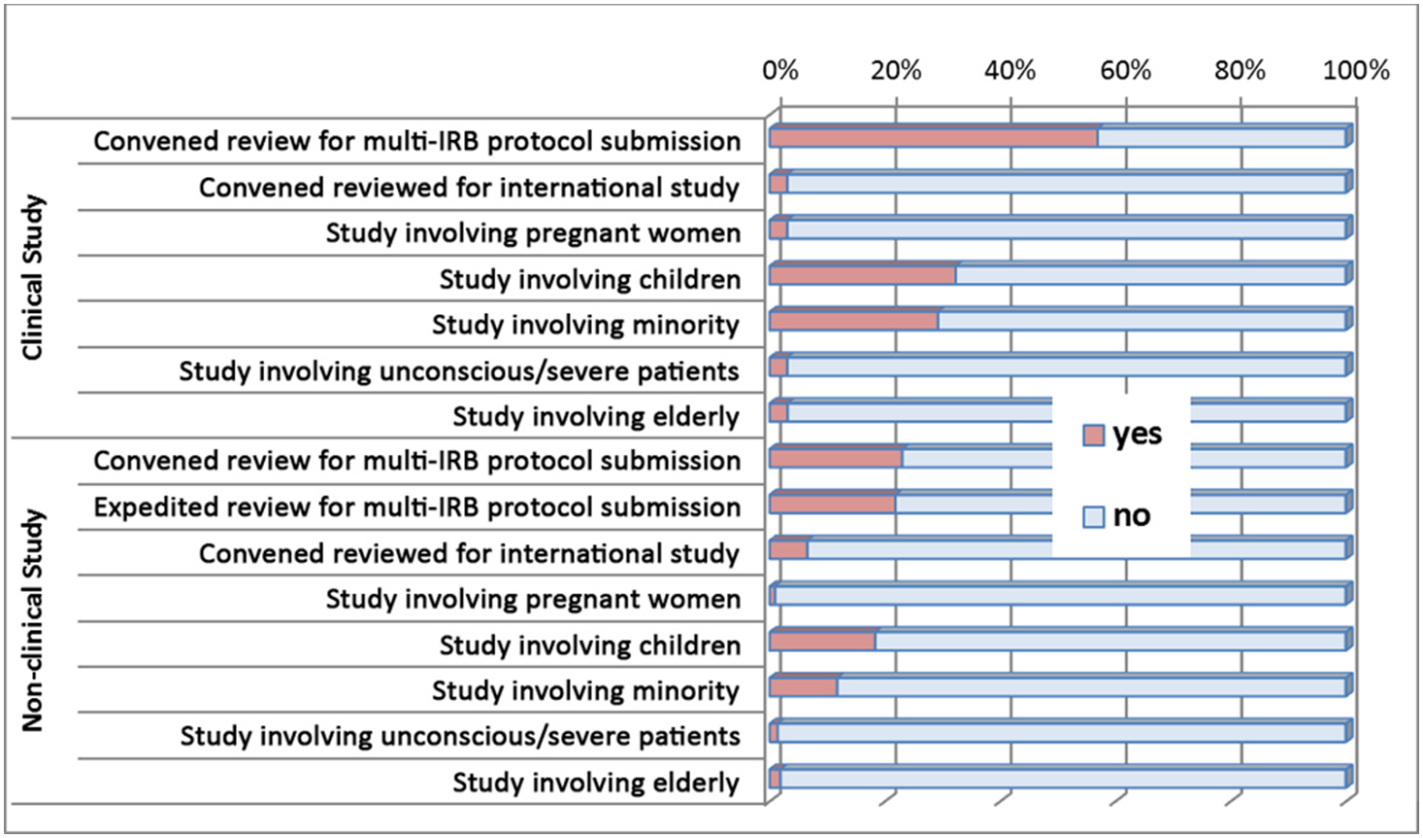
Evaluation of Structure – IRB workload & different types of new non-exempt protocol reviews, 2010–2013.

All new clinical studies were reviewed by a convened IRB, and non-clinical studies by either convened or expedited review. As shown in Figure 2, about 57% of clinical protocols were multi-site studies requiring simultaneous review with other IRBs. About 20% of non-clinical protocols were multi-site studies, and of these, some required a convened review meeting by the FTM-EC, while others could be conducted by expedited review, by primary reviewers only. Expedited reviews are allowed for non-clinical protocols if they have already been reviewed by other IRBs that have a collaborative arrangement with FTM-EC. However, all clinical protocols must be reviewed by a convened meeting. Very few studies, mostly those conducted by international students at the FTM that are conducted in their home countries, still must receive FTM-EC approval. However, these protocols are normally approved after the FTM-EC receives ethical clearance from the host country/countries. Several studies reviewed at FTM-EC required special attention by IRB members, due to the involvement of vulnerable populations.

### IRB Process

Convened IRB review meetings are arranged once per panel per month. More IRB members attend clinical study reviews than non-clinical study reviews. The average (min–max) number of members attending convened meetings for clinical studies (Panel I) was 11 (5–15), while the average for non-clinical studies (Panel II) was 7 (5–10). Average meeting duration was 3 (2–6) hours. The FTM-EC performs an annual monitoring study-site visit for informed quality-assurance review of selected approved studies. Each panel selects a study considered to inhere high risk and/or other characteristics of ethical interest, and then informs the principal investigator, requesting internal monitoring of study activities. In addition, the FTM-EC arranges annual training courses on Good Clinical Practice and Human Protection Studies for the research community.

One of the main concerns of the IRB process is the timeliness of protocol review. It should be noted that no study received exemption from review at the FTM-EC during the 45-month study period. As shown in Table 3, for new non-exempt protocols that required a convened meeting, the number of days from protocol submission to first investigator’s notification (for both clinical and non-clinical studies) was about 30 days. While no expedited review was allotted for clinical studies, for expedited reviews of non-clinical studies, the number of days from protocol submission to first investigator’s notification was about 18 days. The total number of days from submission to final approval for convened-IRB reviews (clinical and non-clinical protocols) decreased over the duration of the current study. For clinical studies, the average and median total numbers were 77 and 85 days. For non-clinical protocol reviews, they were 70 and 60 days, respectively, for convened IRB, and 29 and 25 days for expedited reviews. 50% of new clinical studies and 70% of new non-clinical studies were asked for revisions once. Revisions were requested for only a few studies >2 times. For continuing/amended clinical protocols, the average total days from submission to final approval was about 40 for convened-IRB meetings and <20 days for expedited reviews. The average total days from submission to final approval for continuing/amended non-clinical protocols was <10 for expedited reviews.

**Table 3:** Evaluation of Process – Timeliness of protocol review, 2010–2013.

Metrics		Clinical studies (Panel I)				Non-clinical studies (Panel II)			
		2010	2011	2012	2013	2010	2011	2012	2013
New Non-exempt Studies^a^		N = 18	N = 10	N = 19	N = 13	N = 55	N = 57	N = 57	N = 35
**Days From Protocol Submission to Investigator’s Notification**									
Convened review	Average(SD)	30(11)	30(10)	32(14)	27(4)	44(20)	39(12)	33(9)	31(8)
	Median(Min–Max)	28(12–55)	29(17–45)	28(8–74)	28(20–35)	40(17–100)	39(7–63)	29(22–56)	30(10–46)
Expedited review	Average(SD)					35(39)	19(7)	17(6)	18(7)
	Median(Min–Max)					19(4–155)	18(5–32)	17(7–36)	18(7–28)
**Days From Investigator ‘s First Notification of Investigator’s Last Revision/Clarification** b									
Convened review	Average(SD)	82(68)	51(20)	78(42)	65(28)	30(29)	48(39)	76(51)	39(29)
	Median(Min–Max)	71(11–269)	50(30–90)	69(19–175)	60(25–114)	20(2–109)	43(0–199)	70(17–228)	29(10–111)
Expedited review	Average(SD)					18(21)	23(16)	35(49)	20(19)
	Median(Min–Max)					13(5–76)	22(2–58)	15(1–195)	13(1–55)
**Total Days From Submission to Final Approval**									
Convened review	Average(SD)	111(66)	76(32)	102(48)	77(38)	74(37)	82(43)	96(55)	70(31)
	Median(Min–Max)	101(35–288)	71(21–135)	93(31–205)	85(21–139)	67(31–177)	79(8–238)	97(27–267)	60(30–157)
Expedited review	Average(SD)					47(56)	34(21)	46(49)	29(21)
	Median(Min–Max)					30(4–231)	29(5–84)	32(7–226)	25(7–83)
**Numbers of Times for Revision of A New Study Submission** C		**N = 19**	**N = 12**	**N = 21**	**N = 13**	**N = 59**	**N = 60**	**N = 58**	**N = 36**
None	n (%)	0(0%)	2(17%)	2(10%)	3(23%)	8(13%)	12(20%)	11(19%)	8(22%)
1 time	n (%)	12(63%)	7(58%)	10(47%)	6(46%)	47(80%)	37(62%)	40(69%)	27(75%)
2 times	n (%)	2(11%)	3(25%)	7(33%)	4(31%)	3(5%)	11(18%)	7(12%)	1(3%)
>2 times	n (%)	5(26%)	0(0%)	2(10%)	0(0%)	1(2%)	0(0%)	0(0%)	0(0%)
**Amended Continuing Study** d		**N = 27**	**N = 18**	**N = 23**	**N = 4**	**N = 8**	**N = 6**	**N = 5**	**N = 7**
**Total Days From Submission of Amended Continuing Study to Final Approval**									
Convened review	Average(SD)	35(33)	45(21)	45(19)	-	13(−)	33(18)	72(−)	-
	Median(Min–Max)	16(16–73)	56(21–57)	48(22–63)	-	13(13−)	29(17–63)	72(72−)	-
Expedited review	Average(SD)	24(26)	27(19)	17(10)	19(20)	10(5)	8(−)	6(4)	6(3)
	Median(Min–Max)	17(3–128)	22(9–81)	13(5–47)	11(7–49)	8(4–19)	8(8−)	6(1–11)	7(2–10)

Note:

a Excludes 14 studies in 2010–2012 (6 withdrawn by investigator due to internal study team matters; 8 terminated by the Ethics Committee due to very long delay/non-response after notification).

b Excludes 41 studies that required no revision.

C Includes all new non-exempt study submissions.

d Some studies were amended more than once.

### IRB Outcomes

**Figure 3:**
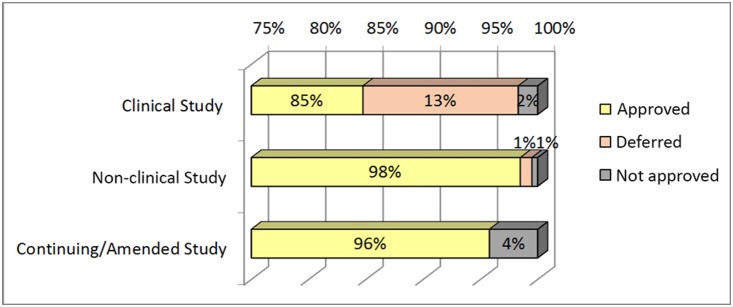
Evaluation of Outcome - Decision on new non-exempt and continuing/amended protocols reviewed by FTM-EC, 2010–2013. *Note: Excludes 7 studies that are pending decision outcome at data cutoff, and 13 studies withdrawn by PI (for various reasons) or by EC (due to long non-response period).*

Most clinical studies (85%) and nearly all non-clinical studies (98%) were approved, with requests to revise some matters. About 13% of clinical studies and 1% of non-clinical studies were deferred, and 2% of clinical and 1% of non-clinical studies were not approved. Almost all amended protocols were approved, with 4% deferred or not approved (Figure 3).

The matters requiring protocol revision for new non-exempt studies revolved around several major ethical points, about which researchers were notified (see Table 4). For both clinical and non-clinical protocols, very few studies were asked about the scientific merit of the study. Over the 45 months of the study, there were requests for clarification of research objectives and study design, and explanations for balancing risks/benefits. Over 90% of clinical studies and 80% of non-clinical studies received comments on research methodology, which covered various ethical issues. About 50% of clinical and 20% of non-clinical studies received comments on sample size, and 23% of clinical studies and 41% of non-clinical studies on confidentiality/privacy. For both types of study, 61% were asked to revise the inclusion-exclusion criteria, 36% the recruitment process, 63% on the specimens required for the study, and 15% on statistical and data-analysis issues. About 20% of clinical studies and 35% of non-clinical studies were asked to revise their protocols due to confidentiality and privacy management issues. In contrast to 90% of clinical studies, 50% of non-clinical studies were required to revise the informed consent process. About 40% received requests to provide documentation, including case record forms and other documents required by the studies (material transfer agreements, investigator brochures, etc.). Few studies received comments on facilities and budgeting. On average, compensation adjustments were requested for 30% of studies.

**Table 4:** Evaluation of Outcomes - Ethical issues notified to researcher.

Metrics	Clinical studies(Panel I)				Non-clinical studies (Panel II)			
	2010	2011	2012	2013	2010	2011	2012	2013
New Non-exempt Studies	N = 19	N = 12	N = 21	N = 13	N = 59	N = 60	N = 58	N = 36
**Numbers of New Non-exempt Studies with Issues Requested for Revisions**								
*(Note: A study may have more than one issue (as shown on IRB initial review form & meeting consensus)*								
Research Question (Scientific merit)	0(0%)	1(8%)	1(5%)	4(31%)	0(0%)	0(0%)	1(2%)	1(3%)
Objectives	3(16%)	2(17%)	5(24%)	6(46%)	10(17%)	14(23%)	9(16%)	0(0%)
Risk & benefit	6(32%)	11(92%)	15(71%)	5(38%)	13(22%)	13(22%)	22(38%)	10(28%)
Study Design	4(21%)	7(58%)	6(29%)	6(46%)	7(12%)	14(23%)	9(16%)	11(31%)
Research Methodology	19(100%)	11(92%)	19(90%)	12(92%)	51(86%)	53(88%)	48(83%)	29(81%)
*-Sample size*	9(47%)	3(25%)	9(43%)	8(62%)	12(20%)	18(30%)	8(14%)	6(17%)
*-Inclusion-exclusion criteria*	12(63%)	7(58%)	10(48%)	9(69%)	34(58%)	43(72%)	33(57%)	25(69%)
*-Recruitment process*	7(37%)	4(33%)	4(19%)	8(62%)	15(25%)	18(30%)	27(47%)	17(47%)
*-Specimen/data collection (amount & procedures)*	17(89%)	10(83%)	16(76%)	11(85%)	41(69%)	32(53%)	38(66%)	12(33%)
*-Statistical & data analysis*	2(11%)	2(17%)	5(24%)	5(38%)	6(10%)	9(15%)	16(28%)	0(0%)
*-Privacy and confidentiality*	2(11%)	2(17%)	9(43%)	2(15%)	20(34%)	27(45%)	28(48%)	15(42%)
Informed Consent (Document + Process)	19(100%)	12(100%)	18(86%)	12(92%)	28(47%)	35(58%)	29(50%)	20(56%)
*-Participation Information Sheet*	17(89%)	12(100%)	18(86%)	12(92%)	26(44%)	34(57%)	29(50%)	20(56%)
*-Informed Consent Form*	17(89%)	11(92%)	15(71%)	12(92%)	24(41%)	33(55%)	26(44%)	14(39%)
Study Documents	6(32%)	6(50%)	7(33%)	6(46%)	22(37%)	25(42%)	26(45%)	16(44%)
*-Related study documents (investigator brochure, advertisement, etc.)*	3(16%)	3(25%)	6(29%)	4(31%)	10(17%)	18(30%)	20(34%)	8(22%)
*-Case Record Forms*	3(16%)	3(25%)	3(14%)	3(23%)	12(20%)	9(15%)	9(16%)	8(22%)
Research Facilities	0(0%)	0(0%)	0(0%)	4(31%)	0(0%)	0(0%)	0(0%)	0 (0%)
Budgeting	0(0%)	0(0%)	4(19%)	0(0%)	0(0%)	1(2%)	1(2%)	1(3%)
Compensation	7(37%)	4(33%)	10(48%)	9(69%)	12(28%)	14(23%)	20(34%)	10(28%)

### Factors associated with “above target duration” of approval process

The normal target duration from protocol submission to approval was set at 60 days, which was based on the monthly cycle of protocol submissions for the monthly convened meetings. Thus the average number of days from submission to first notification was about 30 days, plus on average another 30 days until final approval (making the total average from submission to approval around 60 days). A period of >60 days was considered “above target duration”.

When examining factors that might be associated with the duration of the approval process, it was hypothesized that, despite any uncontrolled delays, such as the investigators’ sending responses to the EC, the type of study and the ethical issues raised by the IRB could be root causes of approval processes with durations above the target limit.

Of all 264 new non-exempt studies during 2010–2013 (excluding 14 studies withdrawn or terminated before the IRB’s final decision), protocol approval was above the target duration in 49% of cases. As shown in Table 5, 77% of clinical studies were above the target duration, compared with 41% of non-clinical studies (PR: 1.9, 95% CI 1.5–2.3). 61% of multi-site studies (multi-IRB submissions) were above the target duration, compared with 43% of studies not involving such settings (PR: 1.4, 95% CI 1.1–1.8). 62%% of studies involving vulnerable populations were above the target duration compared to 42% of studies not involving such populations (PR: 1.5, 95% CI 1.2–1.9). Different issues raised by the IRB were assessed to determine their association with the duration of the approval process in protocol review. The prevalence ratios were quite high on two IRB notification issues: research methodology (PR: 4.6, 95% CI 1.8–11.7) and the informed consent process (PR: 3.5, 95% CI 2.3–5.2). All other IRB comments on ethical issues were also significantly associated with the duration of approval process above the target limit in protocol review. The only issues not statistically correlated with the above target durations were comments on research questions (scientific merit), study design, research facilities, and budgeting.

**Table 5:** Effects of issues reviewed and notified for revision on “above target duration” in total time from submission to final approval (>60 days).

		All Protocols	
Ethical Issues of the Protocol		Percent of studies withtotal time >60 Days	PR* (95% CI)
**Types of Study**			
Clinical study	Yes	46/60 (77%)	1.9 (1.5–2.3)
	No	83/204 (41%)	1
Multi-IRB submission	Yes	51/83 (61%)	1.4 (1.1–1.8)
	No	78/181 (43%)	1
Include vulnerable population	Yes	56/90 (62%)	1.5 (1.2–1.9)
	No	73/174 (42%)	1
**Ethical issues notified to researchers**			
Research question(scientific merits)	Yes	4/7 (57%)	1.2 (0.6–2.3)
	No	125/257 (49%)	1
Objectives	Yes	26/41 (63%)	1.4 (1.0–1.8)
	No	103/223 (46%)	1
Risk and benefit	Yes	62/88 (70%)	1.9 (1.5–2.3)
	No	67/176 (38%)	1
Study design	Yes	31/55 (56%)	1.2 (0.9–1.5)
	No	98/209 (47%)	1
Research methodology	Yes	125/230 (54%)	4.6 (1.8–11.7)
	No	4/34 (12%)	1
Informed consent process	Yes	109/161 (68%)	3.5 (2.3–5.2)
	No	20/103 (19%)	1
Study documents	Yes	61/106 (58%)	1.3 (1.0–1.7)
	No	68/158 (43%)	1
Research facilities	Yes	3/4 (75%)	1.5 (0.9–2.8)
	No	126/260 (48%)	1
Budgeting	Yes	4/6 (67%)	1.4 (0.8–2.5)
	No	125/258 (48%)	1
Compensation	Yes	53/78 (68%)	1.7 (1.3–2.1)
	No	76/186 (41%)	1

*PR = Prevalence Ratio.

About 77% of clinical studies and 41% of non-clinical studies took >60 days from protocol submission to approval. One of the main factors was delay on the investigator’s part. The time from first notification to approval for clinical studies, non-clinical studies, and expedited review studies, were 62, 40, and 15 days, respectively. However, a few studies during the study period experienced a long delay (>100 days) due to the investigator’s internal management issues, rather than the EC’s issues.

### Benchmarking with accredited institutes

To benchmark FTM-EC performance with other accredited institutes under AAHRPP, some indicators were compared. In terms of IRB staffing, the average number of staff for all organizations accredited by AAHRPP is 16.1, and the average number of staff for organizations with 1–100 active protocols is 4.3. The staff of the Ethics Committee at the Office of Research Services, FTM, is 3. As shown in Table 6, average protocols per full-time equivalent for FTM-EC is 26.3 compared to 9.3 at institutions accredited by AAHRPP. However, the number of protocols in convened meetings at FTM is about one-third of those institutions. The percentage of studies reporting deviations was much less at FTM-EC. For accredited organizations with 1–100 protocols, the average number of studies reporting protocol deviation was 38.5, while at FTM-EC it was only 9. Note that, out of all studies submitted to FTM-EC each year during the study period, about 1/4 were clinical studies, and half of these – mostly large scale or multi-center studies – reported protocol deviations. The number of deviations per study varied.

**Table 6:** Benchmarking FTM-EC performance with selected indicators from AAHRPP-accredited institutes, 2012.

Metrics indicators	AAHRPP*	FTM-EC
**IRB staffing (protocol category 1–100)**		
*-Average # of staff*	4.3	3
*-Average # of (new) protocols*	39.8	79
*-Average # of protocols per FTE*	9.3	26.3
**Median number of all protocols** **overseen by an IRB**	414	99
*-Convened meeting*	136	96
*-Expedited review*	133	3
*-Exemption*	8	0
**Protocol deviations reported** **(protocol category 1–100)**		
*-Average # of protocol deviations*	38.5	9
*-Average # of protocol deviations per 100 protocols*	85.7	-
**IRB review time (days)**		
*-From submission to review of convened IRB*	23.0	Mean/Median: 32/29 Min–Max: 8–74
*-From submission to approval of convened IRB*	44.9	Mean/Median: 95/83 Min–Max: 27–267
*-From submission to review of expedited procedure*	19.7	Mean/Median: 17/17 Min–Max: 7–36
*-From submission to approval of expedited procedure*	30.2	Mean/Median: 46/32 Min–Max: 7–226
**Approval rates**		
*-% IRBs having non-approved protocols*	78.6	-
*-% IRBs having rate of non-approved protocols = 2+%*	10%	non-approved = 1.9%

*AAHRPP has compiled an information database from data supplied by 183 client organizations in 2012, [40].

The review time from submission to first notification of convened IRBs at FTM-EC was about 30 days, compared with 23 days at other accredited institutions. The time from protocol submission to approval under convened IRB at FTM-EC was longer, approximately 90 days, compared with 45 days at other accredited institutions. The time from submission to first notification and to approval for expedited reviews at FTM-EC was similar to that of other AAHRPP-accredited institutions. The review time from submission to first notification at FTM-EC and accredited institutions was about 17 days and 19.7 days respectively, while times from protocol submission to approval were about 32 and 30.2 days, respectively. It should be noted that review times from submission to approval decreased over time at both FTM and AAHRPP-accredited institutions. The FTM-EC can be categorized among the 10% of IRBs with about 2% of studies not receiving approval.

## Discussion

IRB Metrics is a useful tool for IRB self-assessment, to control and prevent errors, to measure efficiency and effectiveness, and to correct and prevent conflicts or potential problems [16], [45]. However, one might argue that metrics usually focus on questions of structure and process, and may not reflect the ethics committees’ actual impact on the practice of research [46]. Several solutions have been suggested in the literature on the research participants’ side (for instance: the IRB outcome assessment should improve study participants’ understanding of the risk-benefit of studies, help participants’ decisions to participate in research, and change participants’ attitudes about research), and on the researchers’ side (for example: reduce the risk factor in research, and ensure that the IRB’s guidance to researchers is actually being followed) [46]. The results of this study were based on the IRB Metrics developed and used at FTM-EC, which mainly comprise performance indicators related to the IRB composition and review process. Assessments of review outcomes were also collected, comprising both objective and subjective measures. To assess the IRB’s efficiency, the FTM-EC employs objective outcomes captured quantitatively from IRB Metrics.

In the quality assurance tool developed by OHRP, the first measure concerned general administrative information of the IRB components required for the protection of human research subjects [32]. This is the fundamental marker of IRB quality. One study investigating the issues raised by the Food and Drug Administration (FDA) regarding the quality of independent review board oversight in clinical trials, concluded that IRBs often failed to maintain adequate written standard operating procedures (SOPs) [47]. The IRBs also failed to ensure that they were composed of at least 5 members, with at least one nonscientific member, as well as avoiding any conflicts of interest. Several other studies also reported problems with the IRB structure. One study about the structure and function of IRBs in Africa reported that 11 of 12 IRBs had quorum requirements (half of the committee, or half plus one) for convened meetings; however, two respondents claimed that maintaining the requisite quorum was sometimes problematic due to high member turnover and the busy schedules of members, with resultant difficulties with punctuality and attendance [48]. One commonly noted complaint was the irregularity of local IRBs, resulting in inconsistent practices or policies. This could happen when local university IRBs comprised board members who changed routinely due to faculty rotations [7]. The FTM-EC has not encountered such structural or functional problems, but at times it has been difficult to find an external reviewer with the specific expertise needed for a protocol review. A limitation of the FTM-EC is availability of expertise in specific areas, as well as obtaining independent consultants. To solve this, networking with personnel and specialists from other institutes has been undertaken in order to help identify qualified person(s) needed for certain protocol(s). Most IRB members attended the convened meetings as needed, but there were times where an acting chair had to be selected from IRB members due to a schedule conflict of the nominated chairperson.

IRB workload measurement can be used to assess not only the performance and quality of an IRB, but also its efficiency [32]. In the OHRP quality-assurance tool, some basic workload indicators are: total number of studies reviewed, approximate average duration of an IRB meeting, type of review, having a checklist and minutes of the review, and post-review communication with investigators [32]. The impact of IRB workload on work stress and review quality should be carefully considered, and it has been suggested that having more members to reduce the workload could help obtain optimal IRB efficiency [49]. Previous research also found that IRB time commitment may work well for small research institutions, but can become a significant burden for larger institutions [50]. In assessing the efficiency of IRBs, it has been suggested that organizational interactions are important [51]. IRBs should provide a single point of contact and develop a relationship with researchers to track down needed information, and to enable them to take action quickly, as needed. Even though the numbers of IRB members in both panels of the FTM-EC were sufficient to satisfy quorum requirements, it was noted that the average number of protocols per FTE support staff was high. Three support staff worked on pre-scanning and managing approximately 80 new protocols (excluding continuing/amended protocols and deviations, and adverse-event reporting.

It was reported in literature that most researchers recognize the importance of protecting human subjects from abuse, but some still express their perception that the local IRB system is sometimes an obstacle to conducting research [38], [52]–[55]. As there is a trend towards IRB accreditation, it is important to monitor mechanisms carefully, to improve the review process and to have “evidence-based ethics” that emphasize the importance of data in informing discussions and decision-making about ethical issues raised by the IRB [28], [56], [57]. FTM-EC has thus developed and used IRB Metrics as an information base and methodology to support process quality improvement and communicate effectively with the faculty’s research community.

Studies of IRB performance have revealed that researchers asked for clarification of comments about various ethical issues by FTM-EC. Topics included consent, recruitment, risks and benefits, compensation arrangements, and scientific issues [8], [38]. An empirical study evaluating IRB performance reported that, of the ethical considerations raised consistently by IRB members, 21% were about risk minimization, 57% risk/benefit ratio, 60% equity in subject selection, 54% data monitoring, 25% privacy and confidentiality, 13% protection of vulnerable subjects, 98% informed consent, and 88% about recommended changes in informed consent [50].

A study investigating the matters raised by the FDA in warning letters regarding the quality of IRB oversight in clinical trials also found a failure to ensure that information given to subjects as part of the informed consent process was in accord with human-protection standards [47]. Most studies of IRB quality measurement from the perspective of IRB members, in terms of protocol review processes and deliberations, reported that IRB committees mainly focused on consent forms and processes when completing protocol reviews [38], [52], [54], [55]. As shown in the current study, both FTM-EC panels also had major concerns about participants’ personal information, informed consent forms, and the informed consent process.

Research methodology is another issue discussed and explained to researchers. A previous study conducted by FTM-EC, regarding the approval or non-approval of studies involving minorities, reported that one reason for non-approval was unclear research methodology [58]. The IRB Metrics results also indicate that the importance of research methodology is not research design *per se*, but rather sample size, data and specimen collection, inclusion-exclusion criteria, and confidentiality and privacy. This finding is similar to other IRBs – earlier research suggests that most IRB members elsewhere also reported the issue of proposal review, to clarify elements of the investigator’s research plan [52]. One cause of researcher frustration is that the ethics committee questions the science of the proposal [4]. This remains an issue requiring further consideration among ethicists and ethics committee members, since many researchers believe that this is not the role of the ethics committee. The governance arrangements for research ethics committees (GAFREC) document also supports the view that the ethics committee need not reconsider the quality of the science, which has already been considered by the sponsor and experts in the field [4], [59].

Researchers have also expressed dissatisfaction with delays in studies reviewed by multiple IRBs [2]. In assessing the efficiency of an IRB, average actual turnaround times and processing times are two key indicators suggested in the literature [51], [60]–[62]. Questions to ask IRBs include: How long does it take from submission to board review? How long from the board meeting to final approval of a study? How long does it take for an expedited review? What is the turnaround time for amendments? Can the IRB provide metrics to support its stated turnaround times? [51]. A study on the impact of metrics on human research protection programs found them very useful, pointing out that between 2006 to 2009 the average days from IRB receipt of a study to final approval had decreased from 120 to 88 (for convened meetings). For expedited reviews, these numbers fell from 70 to 15 days, and show some potential for further reduction [17]. Another study, comparing the IRBs of six institutions that review medical education research, reported variability in the timeliness and consistency of IRB reviews [63]. Several university IRBs state timeliness targets in their standard operating procedures and/or use their own IRB metric to set targets for protocol reviews – ranging from 30 to 60 days [21], [22], [64], [65]. At FTM-EC, the duration was approximately 60 days, and when determining the potential root causes of approval durations above this target of 60 days, it was found that notification to researchers regarding research methodology, and informed consent processes/documents, were highly associated with the above target threshold. This suggests that the annual generic workshop on good clinical research practice and human research protection currently arranged by the Office of Research Services may still not completely satisfy the needs of the FTM research community. Plans are underway to increase the focus and effort on training in these topics.

It should be noted that not all research studies should be approved in less time. Quality protocols that comply with ethical standards should be reviewed as quickly as possible, while protocols that contain ethical dilemmas should be addressed through an efficient process, but the length of such process – however efficient – will also be determined by the complexity of the ethical issue that needs to be resolved. As the results of this study suggest, more complex, and/or multi-site studies take more time to review. Thus, there could be reasonable arguments that some protocol reviews demand a lengthy process. However, these delays should not be caused by limitations of the IRB process, for instance, lack of staff for the volume of reviews, too much materials or steps in processing such materials, or the limited/unavailability of IRB members, etc. The FTM-EC can thus use analytic results based on its quantitative IRB Metrics to assess their performance and to ensure that the length of approval process was due to genuine ethical concerns that need to be addressed, and not due to lengthy procedural management in protocol submission and revision.

## Limitations of the Study

The main limitation of this study is that it is based on information from only one institution, the Faculty of Tropical Medicine, Mahidol University. It thus cannot represent the IRBs of other institutions in Thailand. However, the IRB Metrics of the FTM-EC was developed incorporating other IRB metrics derived from the AAHRPP and other institutions. The results reflect the same themes and concerns that IRBs and researchers have encountered in other studies. This may help researchers recognize that the quality concerns of IRB functions and operations have a large degree of commonality when international standards on human subject research protection are applied.

## Conclusion

The IRB Metrics has been used to assess the internal process and outcomes of IRB performance at the Office of Research Services of the Faculty of Tropical Medicine, Mahidol University. It was disseminated to communicate with the research community in an annual open-house of the Office. In response to the IRB Metrics, the Faculty arranged an annual self-assessment meeting of IRB members, to review roles and functions, SOPs, work practices, and institutional guidelines. As suggested in the literature, the development and maintenance of positive collaborative relationships *vav* IRB services is a strategy that can help enhance mutual understanding, avert potential conflicts, and benefit researchers, IRB members, and IRB administrators [66]. The FTM-EC is in the process of improving its submission process, as suggested by our researchers’ comments. In addition to arranging annual refresher courses on ethical considerations and human-subject protection, the Office of Research Services will arrange a protocol-writing workshop for the research community, focusing on issues for which FTM-EC commonly requests revisions. As also suggested in other studies, IRBs could improve and expedite their review process by having a pre-review submission process [67]; the Faculty administration has designed a plan to appoint a Pre-review Committee to assist researchers with protocol preparation, before submission. In benchmarking against the metrics of AAHRPP and other institutions, the FTM-EC aims to reduce the time from submission to approval from 60 to 45 days, during 2014. In conclusion, objective and subjective quality assessment provides a valuable opportunity for the institution to evaluate and improve its IRB review and deliberation processes, and to strengthen relationships and reduce conflicts with researchers, which in turn has a direct positive impact for all involved in the conduct of ethical human-subject research.
